# Pathophysiological Consequences of KATP Channel Overactivity and Pharmacological Response to Glibenclamide in Skeletal Muscle of a Murine Model of Cantù Syndrome

**DOI:** 10.3389/fphar.2020.604885

**Published:** 2020-11-30

**Authors:** Rosa Scala, Fatima Maqoud, Nicola Zizzo, Antonietta Mele, Giulia Maria Camerino, Francesco Alfredo Zito, Girolamo Ranieri, Conor McClenaghan, Theresa M. Harter, Colin G. Nichols, Domenico Tricarico

**Affiliations:** ^1^ Section of Pharmacology, Department of Pharmacy–Pharmaceutical Sciences, University of Bari “Aldo Moro”, Bari, Italy; ^2^ Section of Veterinary Pathology and Comparative Oncology, Department of Veterinary Medicine, University of Bari “Aldo Moro”, Valenzano, Italy; ^3^ Interventional and Medical Oncology Unit, Department of Pathology National Cancer Research Centre, IRCCS Istituto Tumori Giovanni Paolo II, Bari, Italy; ^4^ Department of Cell Biology and Physiology, and Center for the Investigation of Membrane Excitability Diseases, Washington University School of Medicine, St. Louis, MO, United States

**Keywords:** Cantù syndrome, rare disease, ATP-sensitive potassium channel, skeletal muscle, glibenclamide, patch-clamp, histopathology

## Abstract

Cantù syndrome (CS) arises from mutations in *ABCC9* and *KCNJ8* genes that lead to gain of function (GOF) of ATP-sensitive potassium (KATP) channels containing SUR2A and Kir6.1 subunits, respectively, of KATP channels. Pathological consequences of CS have been reported for cardiac and smooth muscle cells but consequences in skeletal muscle are unknown. Children with CS show muscle hypotonia and adult manifest fatigability. We analyzed muscle properties of Kir6.1[V65M] CS mice, by measurements of forelimb strength and ultrasonography of hind-limb muscles, as well as assessing KATP channel properties in native *Flexor digitorum brevis* (FDB) and *Soleus* (SOL) fibers by the patch-clamp technique in parallel with histopathological, immunohistochemical and Polymerase Chain Reaction (PCR) analysis. Forelimb strength was lower in Kir6.1^wt/VM^ mice than in WT mice. Also, a significant enhancement of echodensity was observed in hind-limb muscles of Kir6.1^wt/VM^ mice relative to WT, suggesting the presence of fibrous tissue. There was a higher KATP channel current amplitude in Kir6.1^wt/VM^ FDB fibers relative to WT and a reduced response to glibenclamide. The IC_50_ of glibenclamide to block KATP channels in FDB fibers was 1.3 ± 0.2 × 10^−7^ M in WT and 1.2 ± 0.1 × 10^−6^ M in Kir6.1^wt/VM^ mice, respectively; and it was 1.2 ± 0.4 × 10^−7^ M in SOL WT fibers but not measurable in Kir6.1^wt/VM^ fibers. The sensitivity of the KATP channel to MgATP was not modified in Kir6.1^wt/VM^ fibers. Histopathological/immunohistochemical analysis of SOL revealed degeneration plus regressive-necrotic lesions with regeneration, and up-regulation of Atrogin-1, MuRF1, and BNIP3 mRNA/proteins in Kir6.1^wt/VM^ mice. Kir6.1^wt/VM^ mutation in skeletal muscle leads to changes of the KATP channel response to glibenclamide in FDB and SOL fibers, and it is associated with histopathological and gene expression changes in slow-twitch muscle, suggesting marked atrophy and autophagy.

## Introduction

Cantù syndrome (CS, OMIM#23985, also known as hypertrichotic osteochondrodysplasia) is a rare autosomal dominant inheritance, multi-organ condition (Cantú et al., 1982), characterized by cardiomegaly, vascular dilation, and low blood pressure (Grange et al., 2006; Scurr et al., 2011; Brownstein et al., 2013; Nichols et al., 2013) together with hypertrichosis and skeletal malformations. Neuromuscular symptoms have been observed in some patients (Leon Guerrero et al., 2016). The molecular basis of CS is now recognized to be gain of function (GOF) mutations in the *ABCC9* and *KCNJ8* genes, which encode the regulatory sulfonylurea receptor SUR2 (*ABCC9*) and pore-forming Kir6.1 (*KCNJ8*) subunits of ATP-sensitive K^+^ (KATP) channels (Harakalova et al., 2012; Cooper et al., 2014). To date, ∼70 individuals with CS, associated with >30 missense *ABCC9* or *KCNJ8* mutations have been reported in the literature (Grange et al., 2019). All characterized mutations resulted in enhanced activity of recombinant KATP channels when expressed in heterologous expression systems that arises from the reduced sensitivity to ATP (Cooper et al., 2015; McClenaghan et al., 2018).

Patients with CS have a complex phenotype from birth. Some children with CS show muscle hypotonia leading to delays in the development of motor skills such as sitting, standing, and walking (https://ghr.nlm.nih.gov/condition/cantu-syndrome#definition). Also, adult CS patients tend to have a muscular appearance, caused by an increase in muscle size, although masked by edema when older, and self-report fatigability. KATP channels are present in the membrane of skeletal muscle fibers where they are responsible for unique feedback between muscle cell metabolism and electrical activity (Flagg et al., 2010). In resting skeletal muscle fibers, these sarco-KATP channels are mostly closed and contribute little to the resting membrane potential (Tricarico et al., 1997). Activation of sarco-KATP channels occurs as a response to metabolic stress and reduced ATP/ADP ratio, leading to a reduction in action potential duration (Tricarico et al., 2016a); in so doing, they contribute to the decline in skeletal muscle excitability and force production during prolonged repetitive stimulation and fatigue (Pedersen et al., 2009). Therefore, KATP channels play a protective role in skeletal muscle, helping to preserve structural integrity, avoiding fiber damage caused by intense exercise, buffering ATP levels during fatigue and tetanus, contributing to glucose uptake regulation, and Ca^2+^ handling. This interpretation is supported by the finding that pinacidil, an opener of KATP channels, increases the rate of fatigue in *Extensor digitorum longus* and *Soleus* muscles of wild-type mice (Tricarico et al., 2016b). On the other hand, reduction of sarco-KATP channel activity is one mechanism of the primary and secondary forms of hypokalemic periodic paralysis, transient weakness and hypokalemia (Tricarico et al., 1999a; Tricarico et al., 1999b). Sarco-KATP channels are predominantly composed of Kir6.2/SUR2A subunits, but we have documented the existence in skeletal muscle of hybrid assemblies of other subunits organized as heteromeric complexes (Tricarico et al., 2006).

CRISPR/Cas9–modified mice, in which CS-associated single nucleotide mutations have been introduced into native *KCNJ8* (Kir6.1[V65M]) or *ABCC9* (SUR2[A478V]) loci provide tractable animal models in which to understand cellular mechanisms and organ consequences of CS (Huang et al., 2018), and this has led to the recognition of the role of channel overactivity in vascular smooth muscle in generating the cardiovascular phenotype CS (McClenaghan et al., 2020). The majority of studies investigating KATP channel in skeletal muscle have either used non-selective pharmacological modulators (glibenclamide/pinacidil/diaxoxide) or knock-out of Kir6.2. GOF mutation of *KCNJ8* gene is found to be responsible for the most severe CS phenotype both in humans and animal models. Even so, the consequence of CS mutations in skeletal muscle and the molecular mechanisms responsible for exercise intolerance and muscle fatigability in CS patients appear so far uninvestigated. In this work, therefore, we evaluated the effects induced by the Kir6.1[V65M] CS mutation on skeletal muscle from heterozygous Kir6.1[V65M] (Kir6.1^wt/VM^) and wild type (WT) mice, by combining *in vivo* and *ex vivo* experiments. We investigated the biophysical and pharmacological properties of KATP channels in fast-twitch *Flexor digitorum brevis* (FDB) and slow-twitch *Soleus* (SOL) muscle fibers by patch-clamp technique. The primary endpoint was the change in the KATP channel current recorded in excised macro-patches from skeletal muscle fibers of Kir6.1[V65M] (Kir6.1^wt/VM^) and wild type (WT) mice, and response to glibenclamide. Secondary endpoints were forelimb strength measurements and ultrasonography evaluation of hind limb muscles to assess functional and morphological consequences. Polymerase Chain Reaction (PCR) analysis of gene expression, as well as histopathological and immunohistochemistry evaluations in different muscle types, were performed to understand the cellular origins of these features.

## Materials and Methods

### Animal Care

Novel knock-in Kir6.1[V65M] mice, resembling the human Cantù syndrome (CS), were generated through CRISPR/Cas9 gene editing and genotyped (Huang et al., 2018) at Washington University, Saint Louis, USA, and then transferred to Italy. Kir6.1wt/VM mice (*N* mice = 4) and wild type (WT) mice (*N* mice = 4) were maintained two to four per cage at the Stabulario of the Dipartimento di Farmacia-Scienze del Farmaco, University of Bari, Italy, under the supervision of the veterinary officer according to D.lgs. 26/2014. Experiments were performed on male mice since no evidence of gender differences currently exist in CS (Grange et al., 2019). The temperature of the laboratory was maintained at 22 ± 1°C, with a relative humidity of 50 ± 5%, and under 12:12 light/dark cycles; the animals were provided and maintained on a standard laboratory diet and water *ad libitum.*

### Ethical Statements

Animal care and all experimental protocols are in agreement with the European Directive 2010/63/EU on Animal Protection Used for Scientific Experiments, and the Washington University School of Medicine Institutional Animal Care and Use Committee, and were approved by the Italian Ministry of Health and by the Committee of the University of Bari O.P.B.A (Organization for Animal Health) (prot. 8515-X/10, 30-01-2019). The animal care, protocols, and the sample size (number of mice needed) were calculated at the minimum required to reach the statistical significance based on the primary endpoint according to the 3R “Replace, Reduce, Refine” rules (see below). For minimizing the risk of observer bias and other “experimenter effects,” experiments were conducted “in blind,” meaning that experimenters were unaware, when possible, of the genotype of the animals (Holman et al., 2015).

### *In-vivo* Parameters of Muscle Strength

Evaluation of forelimb strength was performed using a grip strength meter (Columbus Instruments, Columbus, Ohio), according to TREAT–NMD SOPs (De Luca, 2019), by an investigator blinded to mouse genotype. Mice were allowed to grasp a triangular ring connected to a force transducer and then gently pulled away until the grip was broken. The force applied by the animal at this point represents the maximal resistance the animal can use with its forelimbs. For each animal, at least five separate measurements were made within 2 min and averaged. Both the absolute and normalized (to body weight) medium forelimb force values were used for statistical analysis (De Luca et al., 2003).

### Ultrasound Evaluations

Ultrasonography experiments were conducted using the ultra-high frequency ultrasound bio-microscopy system Vevo 2100 (VisualSonics, Toronto, ON, Canada), by an investigator blinded to mouse genotype. Each animal was anesthetized via inhalation (induction with 3% isoflurane and 1.5% O_2_ l/min, then constantly maintained via nose cone at 2-1.5% isoflurane and 1.5% O_2_) and placed on a thermo-statically controlled table (kept at 37°C) equipped with four copper leads which allowed monitoring of heart and respiratory rate. A rectal probe was used to monitor body temperature. The mice were prepared as previously described (Mele et al., 2016; Whitehead et al., 2016; Conte et al., 2017).

Ultrasound acquisitions of the hind limb were performed to evaluate total hind limb volume (in mm^3^), percentage of vascularization (PV%) and hindlimb echodensity by using the MS250 probe. A 3-dimensional (3D) volume scan of the hind limbs was acquired by translating the ultrasound probe parallel to the long axis of the hind limb. Multiple 2D images were acquired at regular intervals in Power Doppler mode. At the end of the procedure, 3D images were reconstructed from previously collected multiple 2D frames and visualized with VisualSonics 3D software (Mele et al., 2019). The software provides us the hindlimb volume value and PV%. Three 2D frames were selected for each mouse and used for echodensity evaluation. In particular, the images were analyzed by using ImageJ^®^ software by creating a gray-scale analysis histogram on the entire outlined hind limb section of constant dimensions of 7392.12 ± 18.45 pixel. For each mouse, hindlimb echodensity was obtained as the main value obtained from 3 images analysis. Echodensity differences were expressed as the percentage change of the mean echodensity of the pixels included in the selected area.

### Animal Sacrifice and Tissue Collection

At the end of the *in vivo* evaluations, the *ex vivo* experimental phase started. No more than 2 animals were sacrificed *per* week, thus a window of 4–5 weeks was necessary to complete these procedures. Animal sacrifice was made by cervical dislocation under ZOLETIL 50/50 (40 mg/kg i.p.) profound anesthesia (Maqoud et al., 2020). Skeletal muscles and hearts were extracted under profound anesthesia. Blot-dried organs were weighed and weights were normalized to the tibia length. All experimental surgical procedures were performed under a sterile cell culture hood in which all the necessary equipment was sterilized to prevent contamination.

### Drugs and Solutions

The normal Ringer solution used during muscles and organ biopsy contained:145 mM NaCl, 5 mM KCl, 1 mM MgCl2, 0.5 mM CaCl2, 5 mM glucose and 10 mM 3-(N-morpholino) propanesulfonate (Mops) sodium salt and was adjusted to pH 7.2 with Mops acid. For inside-out patch experiments on FDB/SOL fibers, the patch-pipette solution contained: 150 mM KCl, 2 mM CaCl2 and 1 mM Mops (pH 7.2); the bath solution contained 150 mM KCl, 5 mM EGTA and 10 mM Mops (pH 7.2). Stock solutions of glibenclamide (Glib) (118.6 mM) were prepared by dissolving the drug in DMSO (Tricarico et al., 2012). DMSO applied at the maximal concentration tested, which was 0.05%, did not affect the channel currents in the absence or the presence of ATP (solvent control).

### Patch-Clamp Experiments on Flexor digitorum brevis and Soleus Fibers

Experiments on FDB/SOL fibers were performed in inside-out configurations by using the standard patch-clamp technique (Tricarico et al., 2008a). Isolated fibers were obtained from FDB and SOL muscles by enzymatic digestion with collagenase (C9697 Sigma, ≈0.5 mg/ml). Channel currents were recorded in excised macro-patches (R = 0.91 ± 0.07 MΩ) during voltage steps from a holding potential of 0 mV to −60 mV (Vm) immediately after excision, at 20–22°C. Currents were recorded at a 1-kHz sampling rate (filter 0.2 kHz) by using an Axopatch-1D amplifier equipped with a CV-4 headstage (Molecular Devices, CA) (Scala et al., 2019). The current amplitude was measured using Clampfit 10.0. Patch pipettes were pulled from borosilicate glass capillaries (Glass type 8250, King, USA) and fire-polished. Macro-patches containing significant voltage-dependent K+ channels or other Kir channels or showing marked loss of channel currents during the time of observation were excluded from the analysis. No correction for liquid junction potential was made, estimated to be <1.9 mV in our experimental conditions.

### Fiber Survival Evaluation

Evaluation of the morphological parameters of FDB fibers was performed seeding fibers in the culture medium (DMEM supplemented with 10% fetal bovine serum, 1% L-glutamine and 1% penicillin-streptomycin), at 37°C (Mele et al., 2012), by an investigator blinded to mouse genotype. Before analysis, isolated fibers were equilibrated in the culture medium for at least 30 min at 37°C. Fiber morphology was evaluated using a Nikon TMS Inverted Microscope 4x magnification. Dead fibers were defined as cells showing marked changes of ≥40% in morphological parameters such as length and diameter within 24 h from the excision. The appearance of multiple sarcolemma blebs preceded cellular death.

### Histopathological Analysis

After animal sacrifice, tissue samples were embedded in OCT tissue-freezing medium, frozen in liquid nitrogen, and stored at −80°C until analysis. Samples were fixed with 10% animal buffered formalin for a minimum period of 48 h. Tissues were incorporated into the paraffin, sections were cut to 5–10 μm and stained with standard techniques with hematoxylin and eosin (H.E.), Mallory trichrome stain, and periodic acid-Schiff (PAS). Other specimens stored at −80 were used for ATPase and Succinodehydrogenase stains in cryo-section. Muscles were cross-sectioned using a microtome. Digital images were taken from the cross-section at 10-100x magnification to evaluate muscle fiber morphology and to determine fiber cross-sectional area (CSA) measures. Images from 20 random fields were acquired for the ten stained sections of each specimen using a D 4000 Leica DMLS microscope equipped with a camera and image analyzer NIS elementes-BR-Nikon. Section analysis and the CSA evaluation of the fibers was performed by QWin software (Leica). Of the subjects sacrificed, a gross necropsy of all organs was also carried out (Trapani et al., 2017). Examination of cellular morphology as well as of intracellular structures was conducted and the severity of the observed lesions was assessed (Gibson-Corley et al., 2013), by animal pathologists blinded to mouse genotype.

### Immunohistochemistry

Isolated organs were immunohistochemically stained according to the labeled streptavidin avidin-biotin (LSAB) method using Autostainer Link 48 Immunohistochemistry Staining System (Agilent Technology). The slides were controlled by protocols in the DakoLink software. Tissue sections were cut (4 µm thick), placed on poly-L-lysine-coated glass slides, and, subsequently, deparaffinized in xylene and dehydrated, and IHC slides automatically mounted and cover-slip applied, after staining and dehydration. To detect anti-genes, the sections were immersed in citrate buffer (0.1, pH 0.6), for 30 min with 0.3% hydrogen peroxide, then in methanol for 12 min to quench endogenous peroxidase activity. After washing three times for 5 min each with phosphate-buffered saline (PBS), the sections were blocked by soaking for 20 min at room temperature in PBS containing 1% bovine serum albumin. The blocked sections were incubated overnight at 48°C anti-mouse primary antibodies for NFκB p50 (E-10): sc-8414; MAFbx (F-9): sc-166806; caspase-3 (E-8): sc-7272; BNIP-3 (ANa40): sc-56167 (Santa Cruz Biotechnology, Inc.) diluted 1/100 for 40 min then thoroughly washed in 0.05 M buffered with Tris saline (pH = 7.6) and incubated with streptavidin-peroxidase (Dako, 1: 100) for 40 min. 3, 3- diaminobenzidine (DAB) (Dako Glostrup, Denmark) was used as the chromogen; to counteract the core of Gill'S hematoxylin (Polysciences, Warrington, PA) after sections have been dehydrated and assembled (Zizzo et al., 2019). Experiments were performed by an investigator blinded to mouse genotype.

### Polymerase Chain Reaction

Total RNA was isolated and purified from entire FDB and SOL muscles with Trizol reagent (Invitrogen Life Technologies) and quantified using a spectrophotometer (ND-1000 Nano-Drop, Thermo Scientific). PCR amplification was achieved using PCR Master Mix (Promega) (Dinardo et al., 2012). PCR cycles consisted of denaturation at 95 °C for 1 min, annealing segment at 58 °C for 1 min, and extension at 72 °C for 1 min, repeated for 30 cycles. Amplified PCR products were separated on 1% agarose gel. Primer sequences are reported in Table 1. The data collection and analysis were performed according to the MIQE (Bustin et al., 2009). All experiments were performed in duplicates per muscle and duplicates per genotypes.

**TABLE 1:** Primer sequences for PCR analysis

		Sequence (5' - 3')
*ABCC8*	F	ATC​ATT​CTG​CTG​GCT​CCT​GT
*ABCC8*	R	CTG​GTC​ATT​TCC​TTC​CTG​CG
*ABCC9*	F	CTG​GTC​CCA​CAT​GTC​TTC​CT
*ABCC9*	R	ATG​CGA​GTC​TGA​AAC​GAT​GC
*KCNJ8*	F	GTA​GAC​CTG​AAG​TGG​CGT​CA
*KCNJ8*	R	GCA​TGG​CGG​CTG​AAA​ATC​A
*KCNJ11*	F	CAC​CTC​CTA​CCT​AGC​TGA​CG
*KCNJ11*	R	ATG​CTA​AAC​TTG​GGC​TTG​GC
*FBXO32*	F	AAG​TCA​CAG​CTC​ACA​TCC​CT
*FBXO32*	R	TGT​TAA​TGT​TGC​CCA​CCA​GC
*TRIM63*	F	AAG​TGA​TCA​TGG​ACC​GGC​A
*TRIM63*	R	AAG​TAG​GCA​CCT​CAC​ACG​TG
*BNIP3*	F	GCT​CCC​AGA​CAC​CAC​AAG​AT
*BNIP3*	R	TGC​GCT​TCG​GGT​GTT​TAA​AA
*CASP3*	F	GAG​CAG​CTT​TGT​GTG​TGT​GA
*CASP3*	R	TGT​CTC​AAT​GCC​ACA​GTC​CA

### Data Analysis and Statistics

Data were collected and analyzed using Excel software (Microsoft Office 2010), Clampfit 10.5 (Molecular Devices), and SigmaPlot 10.0, and statistical results are presented as mean ± SEM unless otherwise indicated. The number of replicates relative to each experimental dataset was reported in the results paragraph and the figure description.

The primary endpoint was the change in the current amplitude recorded in excised patch experiments. The comparisons between groups were performed by using the *t*-test, two tails between two independent means to evaluate variance between groups. The *p*-value was considered statistically significant if <0.05. The number of experimental groups: = 2, WT, and Kir6.1^wt/VM^ mice. The calculation of the sample size was made considering a delta change of the KATP current amplitude = 412 pA and SD of 100 in patch-clamp experiments performed in previous experiments on smooth muscle cells in the transgenic mice Kir6.1^wt/VM^ mice vs WT mice since no data on skeletal muscle cells are so far available for the calculation (Huang et al., 2018). The calculated sample size per genotype was 4, the critical t value of 2.44 with an effect size of 4. The theoretical power of the study for the calculation of the sample was 0.95 in input and remains high in output 0.99 (G * Power 3.1.9.7).

The Student *t*-test was also used to evaluate the significance of differences between the means of two groups for secondary endpoints, *p* values < 0.05 were considered to indicate statistical significance unless otherwise indicated. One WAY ANOVA was used to evaluate intergroup and intragroup variability (*p* < 0.05). The percentage of KATP current inhibition induced by glibenclamide (Glib) was calculated as -(I CTRL- I drug)/(I CTRL-I leak) x 100, where I leak was the current recorded after the application of 5 × 10^−3^ M MgATP on skeletal muscle fibers. The frequency of myofibers positivity was calculated on 50 cells per section (Zizzo et al., 2019).

## Results

### Cantù Kir6.1^wt/VM^ Mice Show Enlarged, but Weaker Skeletal Muscles and Higher Echodensity

**FIGURE 1:**
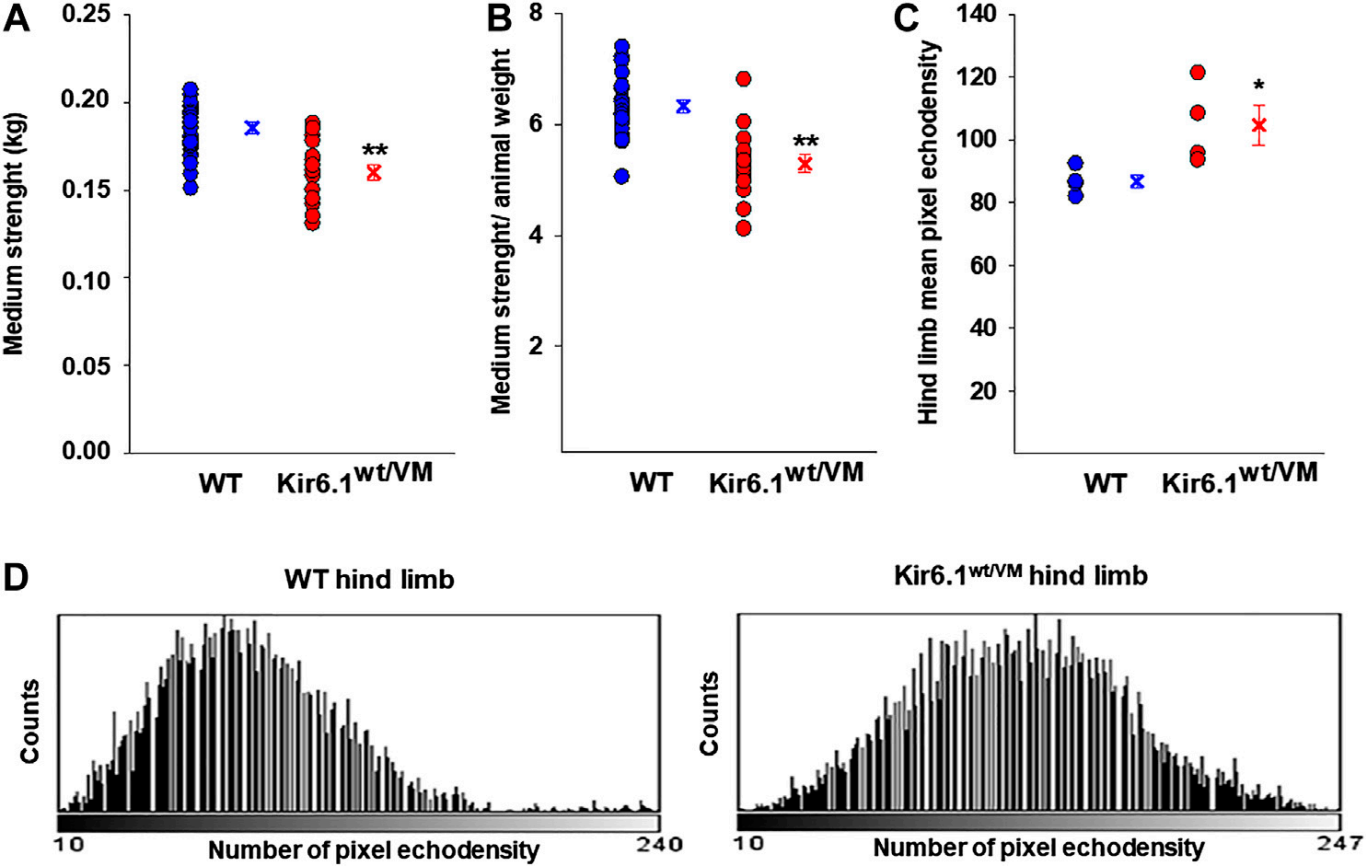
*In vivo* evaluation of skeletal muscle forelimb muscle strength and morphology in WT (*n*. mice = 4) and Kir6.1^wt/VM^ (*n*. mice = 4). Either **(A)** absolute medium forelimb strength and **(B)** forelimb strength normalized for body weight medium are lower in Kir6.1^wt/VM^ mice concerning WT mice. Absolute medium forelimb strength is 0.185 ± 0.003 kg in WT mice (*n*. measurements = 21) and 0.160 ± 0.005 kg in Kir6.1^wt/VM^ mice (*n*. measurements = 20). The forelimb strength normalized for body-weight is 6.326 ± 0.119 in WT mice vs 5.288 ± 0.159 in Kir6.1^wt/VM^ mice. **Data significantly different concerning the control (Student *t* test, p<0.001). **(C)** In an ultrasound evaluation, the mean pixel echodensity in Kir6.1^wt/VM^ hind limb was significantly higher than WT. The mean echodensity of hind limb is 86.78 ± 2.15 (*n*. animals = 4) in WT and 104.77 ± 6.42 (*n* animals = 4) in Kir6.1^wt/VM^ mice, resulting in a significant enhancement of +20.72% of this parameter in Kir6.1^wt/VM^ mice compared to the WT mice. *Data significantly different concerning the control (Student *t* test, p<0.05). Data are presented as individual data points in the vertical point blot and as mean ± SEM. **(D)** Sample representative images of the grey-scale histogram obtained by ImageJ software analysis of the selected hind limb showing a white-shift of the pixel intensity in Kir6.1^wt/VM^ mice concerning the WT mice.

We first assessed the macroscopic properties of skeletal muscle in terms of strength and integrity. At the beginning of the experimental protocol, 33-week-old Kir6.1^wt/VM^ and age-matched WT mice were not significantly different in weight (30.6 ± 1.7 g *n* = 4 Kir6.1^wt/VM^ mice vs 29.5 ± 1.3 g *n* = 4 WT mice, *p* = 0.321), but forelimb grip strength (both absolute and normalized to body weight) was significantly lower in Kir6.1^wt/VM^ mice than in WT mice (Figures 1A,B).

**FIGURE 2:**
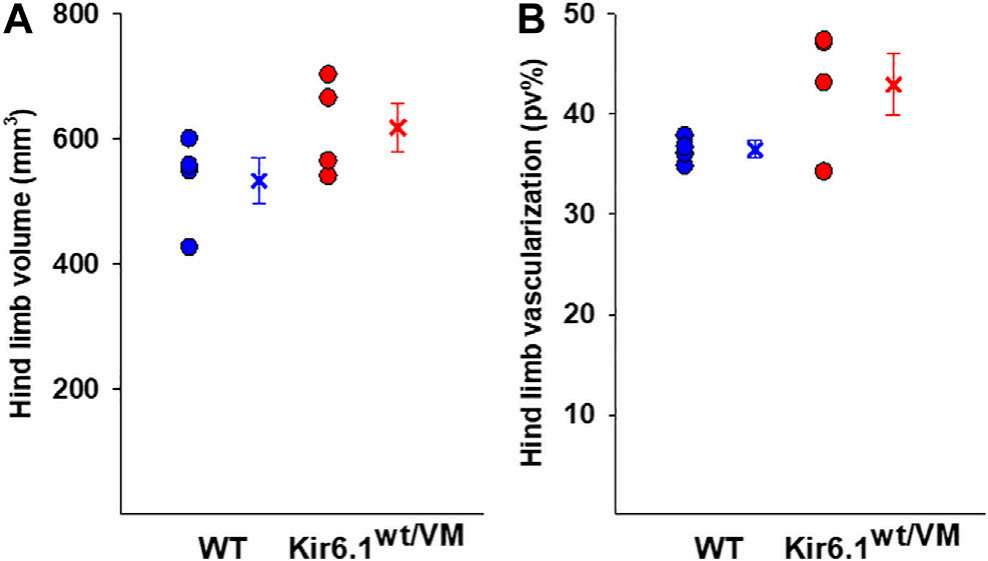
Ultrasound evaluation of hind limb A tendency toward higher hind limb **(A)** volume and **(B)** vascularization was found in Kir6.1^wt/VM^ mice (*n*. mice = 4) concerning the WT (*n*. nice = 4). Data are presented as individual data points in the vertical point blot and as mean ± SEM.

Hind limb muscle morphology was assessed by 3D ultrasound evaluation. Interestingly, hind limb echodensity was ∼20% higher in Kir6.1^wt/VM^ than in WT (Figures 1C,D), reflecting lower muscle mass and fibrotic tissue/fat deposition. Although not significant, there was a trend towards increased hind limb volume and percentage of vascularization (PV%) in Kir6.1^wt/VM^ mice (Figures 2A,B).

**FIGURE 3:**
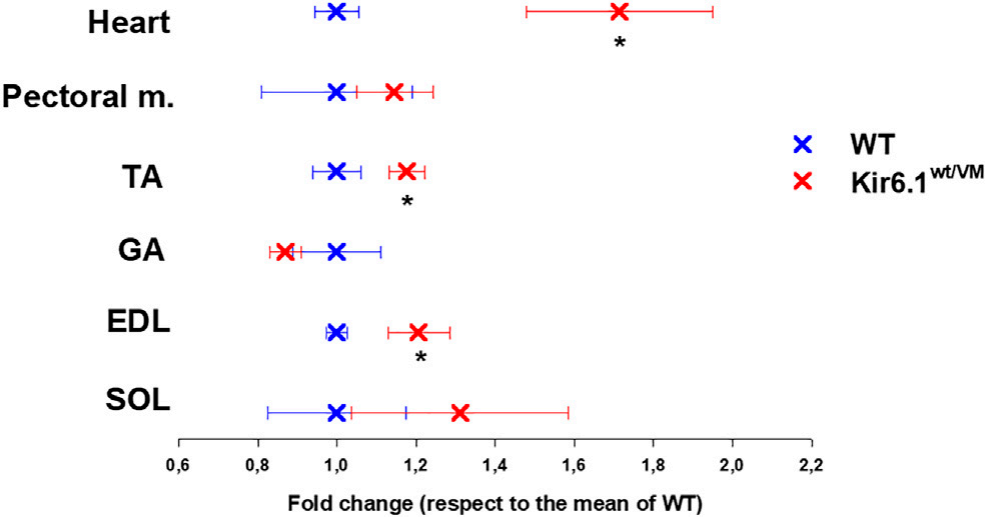
Organ weight differences between WT and Kir6.1^wt/VM^ mice. A tendency toward higher organ weight is observed in Kir6.1^wt/VM^ mice. Values are presented as mean ± SEM (*n*. mice = 4 for WT and Kir6.1^wt/VM^); organ mean weights are normalized by the control mean. No differences were found in terms of weight among right and left muscles. GA, Gastrocnemius; TA, Tibialis anterior; EDL, Extensor digitorum longus; SOL, Soleus; PECTORAL M., Pectoral muscles. *Data significantly different within groups and between groups was evaluated by one WAY ANOVA with calculated F values > 1.3 (*p* < 0.05).

Following sacrifice, individual muscles were isolated and weighed. As previously reported (Huang et al., 2018) there was dramatic cardiomegaly in Kir6.1^wt/VM^ mice, which showed a ∼1.7-fold increase in heart weight as evaluated by one WAY ANOVA (Figure 3). There was also a tendency toward increased skeletal muscle weights in Kir6.1^wt/VM^ mice, including *Soleus* (SOL), and significant increases in *Extensor digitorum longus* (EDL) and *Tibialis anterior* (TA) muscles as evaluated by one WAY ANOVA (Figure 3).

### KATP Channels Show Reduced Sensitivity to Glibenclamide in Cantù Kir6.1^wt/VM^ Muscles

**FIGURE 4:**
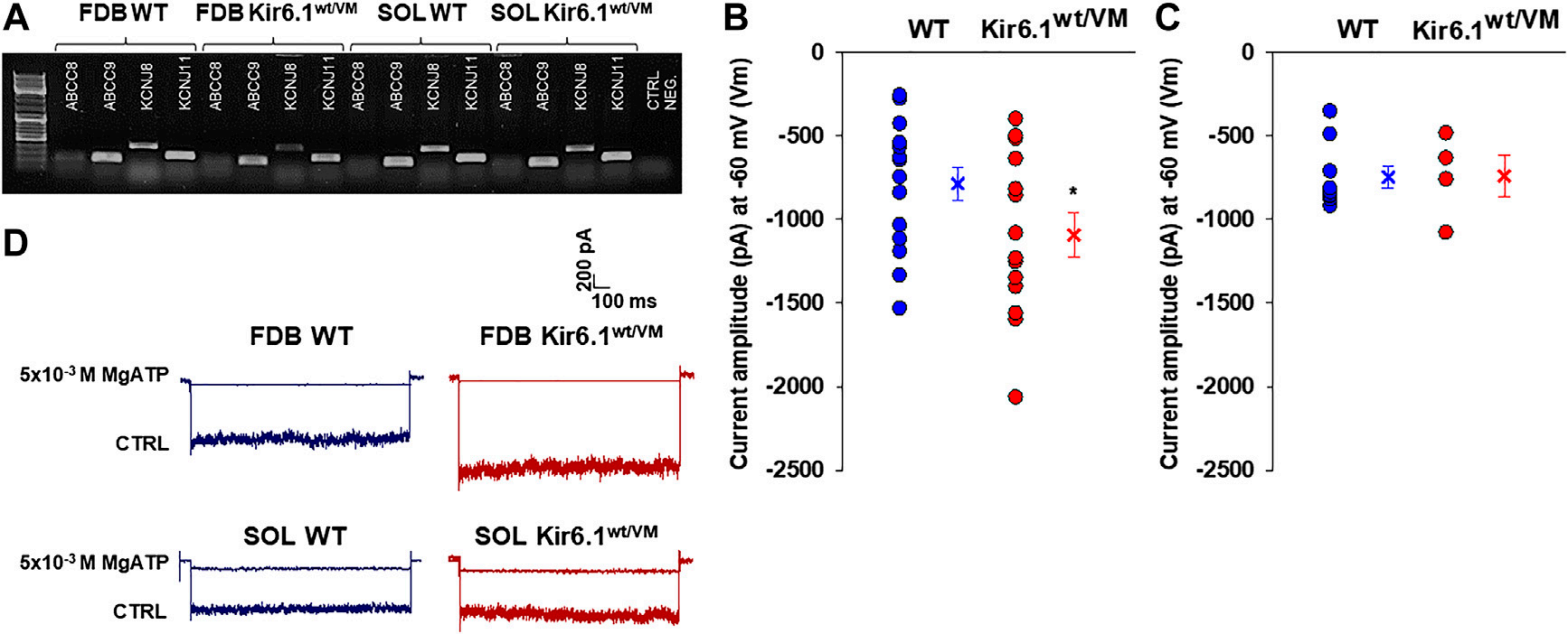
Channel subunits expression and current amplitude distribution in FDB and SOL fibers at physiological membrane voltage of −60 mV (Vm). **(A)** Representative sample gel of PCR analysis in FDB and SOL muscles: high levels of expression of *ABCC9* and *KCNJ11* genes, encoding respectively SUR2 and Kir6.2, were found in both phenotypes. *KCNJ8* gene, encoding Kir6.1 subunit, was found expressed both on FDB and SOL muscles of WT and Kir6.1^wt/VM^ mice. **(B)** In inside out patch-clamp experiments on fast-twitch FDB fibers, the calculated mean was −786.9 ± 98.3 pA for WT (*n*. patches = 15 from ≥ 3 mice) and −1092.6 ± 130.9 pA for Kir6.1^wt/VM^ (*n*. patches = 14 from ≥ 3 mice). (*data are significantly different, Student *t* test, *p* < 0.05) **(C)** In slow-twitch SOL fibers, the calculated mean was −748.9 ± 65.3 pA for WT (*n*. patches = 9 from ≥ 3 mice) and −740.9 ± 126.3 pA for Kir6.1^wt/VM^ (*n*. patches = 7 from ≥ 3 mice). Values of current amplitude are presented as individual data points in the vertical point blot and as mean ± SEM. **(D)** Representative sample traces of the recorded KATP currents in inside-out patch-clamp experiments in FDB and SOL fibers. Higher current amplitude was observed in Kir6.1^wt/VM^ FDB fibers concerning the WT whereas no differences were detected among the two phenotypes in SOL fibers. 5 × 10^−3^ M MgATP completely closed KATP currents.

We confirmed the expression of KATP channel subunits in fast-twitch FDB fibers and slow-twitch SOL fibers by PCR analysis of *KCNJ8, KCNJ11, ABCC8,* and *ABBC9* genes (encoding Kir6.1, Kir6.2, SUR1, and SUR2, respectively), of FDB (*n*. muscles = 2) and SOL (*n*. muscles = 2) whole muscle samples. Specific bands were detected for *KCNJ11* and *ABCC9* in both FDB and SOL (Figure 4A), which is consistent with the prevailing evidence that skeletal muscle KATP channels are composed predominantly of Kir6.2/SUR2 subunits (Tricarico et al., 2006). However, a prominent band for *KCNJ8* was also present in both fast-twitch and slow-twitch muscles, with low expression level in the FDB muscle of Kir6.1^wt/VM^ mice (Figure 4A).

**FIGURE 5:**
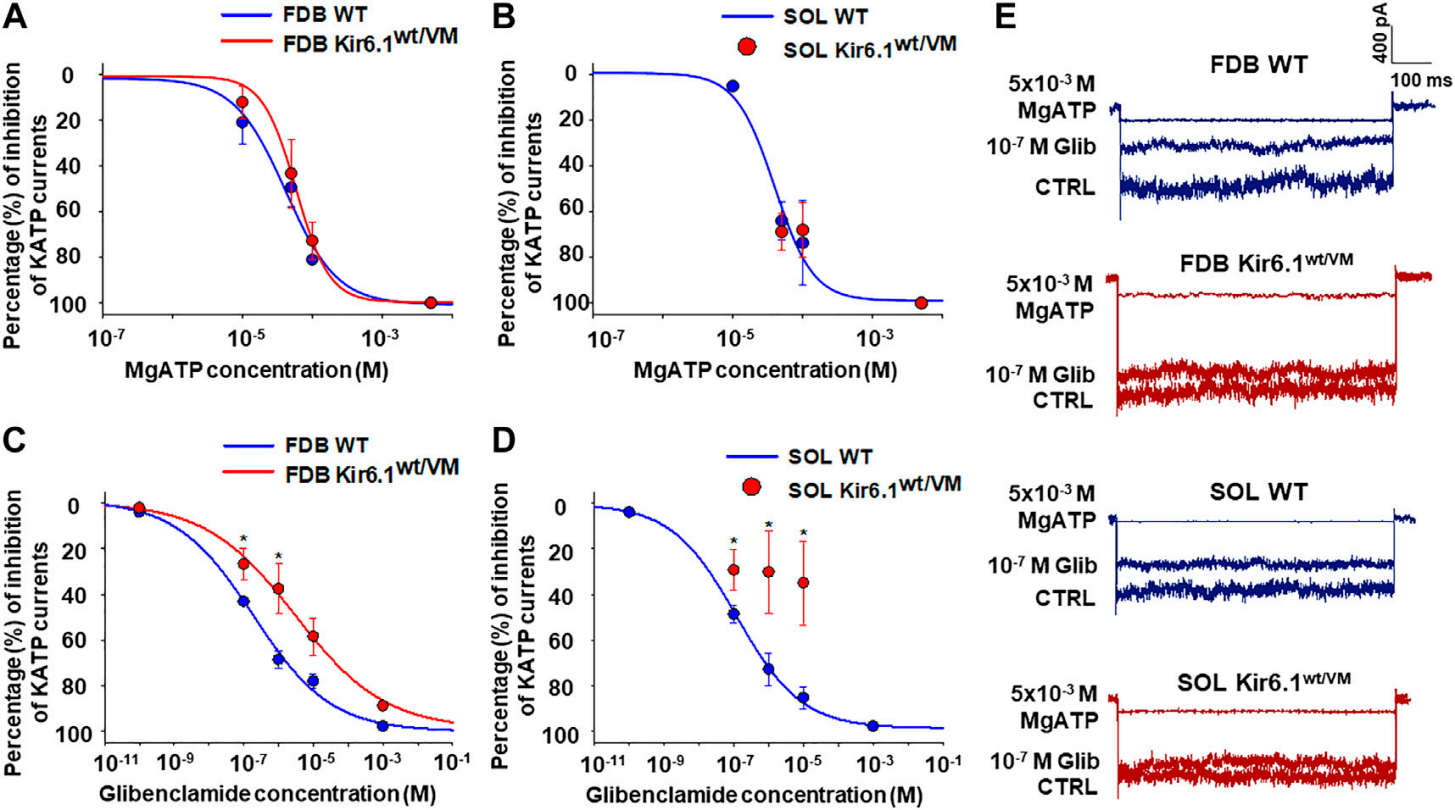
Percentage changes of KATP currents amplitude vs. MgATP or glibenclamide (Glib) concentrations in FDB and SOL muscles. **(A)** A slight rightward shift of MgATP-sensitivity was detected in FDB patches whereas **(B)** no difference in response of the KATP currents to MgATP was observed in Kir6.1^wt/VM^ SOL fibers concerning the WT. **(C)** Reduced sensitivity to Glib was observed in Kir6.1^wt/VM^ FDB fibers concerning the WT. **(D)** A partial inhibitory effect of Glib was observed in Kir6.1^wt/VM^ SOL fibers concerning the WT. **(E)** Sample traces of CTRL currents, 10^−7^ M Glib, and 5 × 10^−3^ M MgATP in WT FDB, Kir6.1^wt/VM^ FDB, WT SOL, and Kir6.1^wt/VM^ SOL. *Data significantly different concerning the control (Student *t* test, *p* < 0.05). Data were fitted using the Hill equation; fitting analysis failed for Kir6.1^wt/VM^ SOL fibers, for which only a few experimental points were collected. Each experimental point represents the mean ± SEM of at least three patches from ≥ 3 mice each.

KATP channel activity was recorded in macro-patches excised from acutely dissociated FDB fibers (*n*. muscles = 4) and SOL fibers (*n*. muscles = 4) using the patch-clamp technique. Mean current density (i.e. maximum current in zero ATP) was ∼1.5-fold higher in Kir6.1^wt/VM^ than WT FDB patches but was not different between genotypes in SOL patches (Figures 4B–D). However, we note that Kir6.1^wt/VM^ SOL fibers showed sudden changes in length and diameters and surface blebs appearance within 2 h after acute isolation, resulting in a low percentage of successful experiments, and potentially reflecting membrane damages and muscle pathology. We assessed the sensitivity of KATP to MgATP, which provides a compound measure of both inhibition by ATP itself and the activator effect of MgATP at the SUR subunit (Reimann et al., 2000; Proks et al., 2014; McClenaghan et al., 2018). There was no clear shift of MgATP-sensitivity of the KATP channels in SOL and FDB patches (Figures 5A,B; Table 2). We tested the KATP channel sensitivity to the KATP channel inhibitor glibenclamide (Glib). In both FDB and SOL patches, there was a significant reduction of Glib-sensitivity, with ∼50% reduction in the inhibitory effect of 10^−7^ M Glib in each muscle (Figures 5C–E; Table 2).

**TABLE 2:** Fitting parameters of the concentration–response relationships of percentage reduction of KATP currents amplitude vs. MgATP or Glib concentrations in FDB and SOL muscles. Values are expressed as the mean ± SEM of at least three replicates, as evaluated by using SigmaPlot 10. *Data significantly different with respect to the WT data (Student *t* test, p<0.05). N.a. indicates experimental conditions for which fitting parameters were not obtained.

Muscle types	MgATP E max (%)		MgATP IC_50_ (M)		MgATP Hill slope	
	WT	Kir6.1^wt/VM^	WT	Kir6.1^wt/VM^	WT	Kir6.1^wt/VM^
FDB	−100.6 ± 4.4%	−99.5 ± 0.4%	4.2 ± 0.8 x 10^−5^	5.8 ± 0.07 x 10^−5^	1.2 ± 0.3	1.8 ± 0.05
SOLEUS	−98.9 ± 4.1%	n.a.	4 ± 0.7 x 10^−5^	n.a.	1.6 ± 0.4	n.a.

	Glib E max (%)		Glib IC_50_ (M)		Glib Hill slope	
	WT	Kir6.1^wt/VM^	WT	Kir6.1^wt/VM^	WT	Kir6.1^wt/VM^
FDB	−97.9 ± 1.3%	−93.7 ± 1%	1.3 ± 0.2 x 10^−7^	*1.2 ± 0.1x10^−6^	0.6 ± 0.01	*0.4 ± 0.01
SOLEUS	−98.8 ± 2.2%	n.a.	1.2 ± 0.4 x 10^−7^	n.a.	0.5 ± 0.1	n.a.

Together, these results indicate that Kir6.1 subunit is functionally present in KATP channels in both SOL and FDB muscles resulting in a marked loss of channel sensitivity to Glib in Kir6.1[V65M] mice.

### Cantù Kir6.1^wt/VM^ Skeletal Muscle Fibers Show Pathologic Atrophy and Low Survivability

**FIGURE 6:**
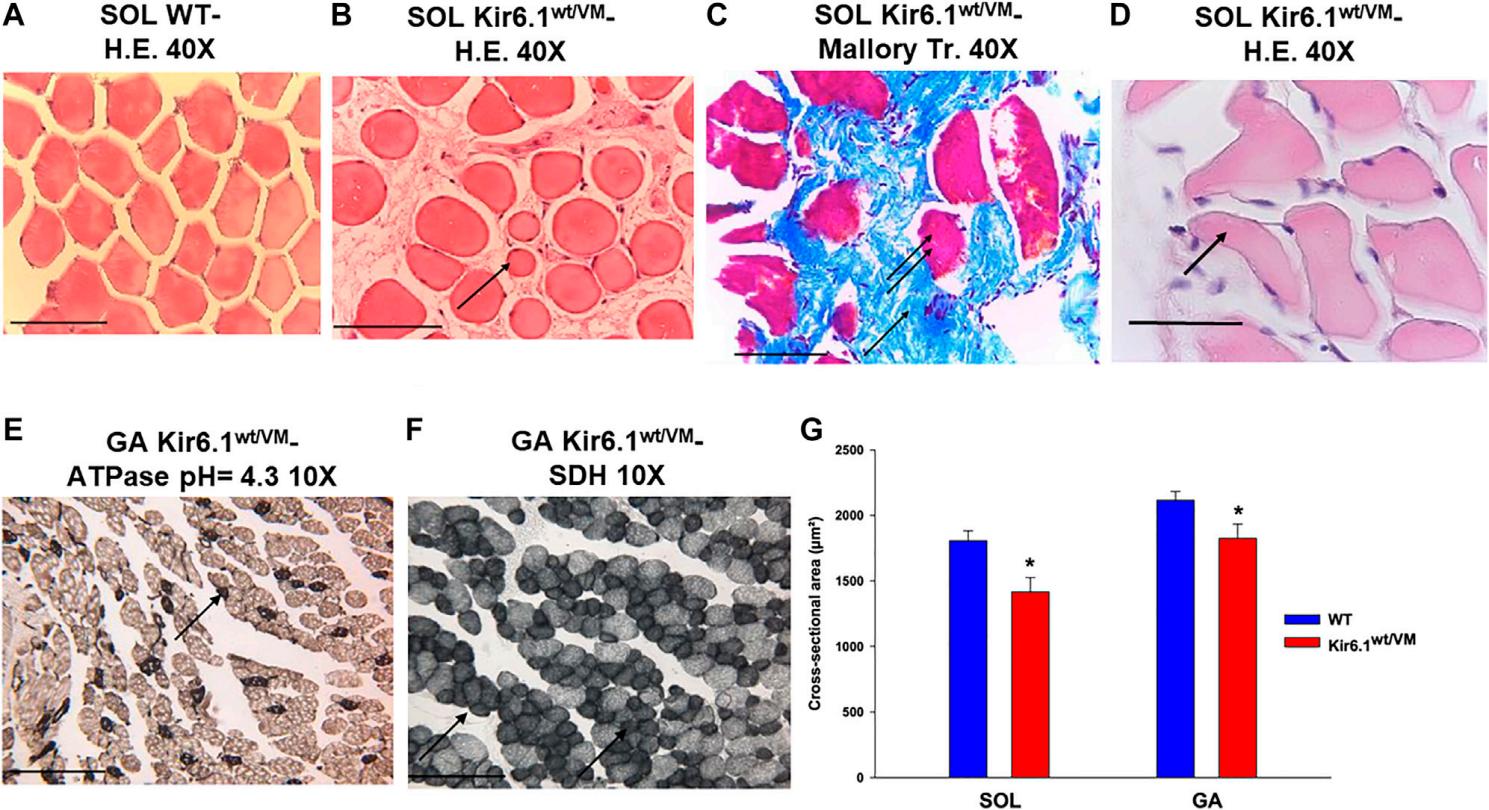
Histological analysis on slow-twitch *Soleus* SOL and fast-twitch *Gastrocnemius* (GA) muscle from WT and Kir6.1^wt/VM^ mice. **(A)** Section of SOL from WT mice shows no sign of damage (H.E 40X, Bar 100 μm) whereas **(B)** atrophic phenomena (arrow) with myofibrils of various sizes grouped according to the zones in small or in large groups in SOL section from Kir6.1^wt/VM^ mice (H.E. 40X, Bar 100 μm). **(C,D)** In the SOL section from Kir6.1^wt/VM^ mice, the myofibrils red-stained (double arrows) consequent to necrotic phenomena can be surrounded by abundant endomysia connective tissues blue stained (arrow) replacing them completely (*C*, Mallory trichrome 40X, Bar 100 μm). **(D)** In the SOL section from Kir6.1^wt/VM^ mice, myofibrils with angular shapes, rounded corners (arrow), and pyknotic nuclei (H.E. 40X, Bar 100 μm) were observed. **(E,F)** In GA sections from Kir6.1^wt/VM^ mice, atrophy in succinodehydrogenase (SDH) positive myofibrils, which appear colored in dark gray sections and stained by acidic ATPase reaction (arrow) (pH = 4.3 10X, Bar 100 μm) was observed. **(G)** Bar chart showing the cross-sectional area (CSA) of slow-twitch SOL and fast-twitch GA muscles in WT and Kir6.1^wt/VM^ mice, as assessed with H.E. coloration. A strong reduction of CSA is observed in both muscles of Kir6.1^wt/VM^ mice; in SOL muscle CSA = 1805.88 ± 76 μm^2^ in WT mice (*n* sections = 58) vs. 1415.18 ± 111 μm^2^ in Kir6.1^wt/VM^ mice (*n* sections = 38); in GA muscle CSA = 2115.98 ± 66 μm^2^ in WT mice (*n* sections = 48) vs. 1825.39 ± 109 μm^2^ in Kir6.1^wt/VM^ mice (*n* sections = 47). Values are expressed as the mean ± SEM. * Data significantly different concerning the WT data (Student *t* test, *p* < 0.05).

**FIGURE 7:**
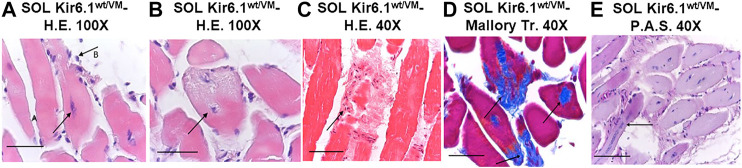
Histological analysis of slow-twitch SOL muscle from Kir6.1^wt/VM^ mice. **(A)** Advanced degeneration characterized by the central nuclei (arrow A) and surrounding inflammatory cellular elements (arrow B) (H.E. 100X, Bar 100 μm), **(B,C)** cell death (*B*, 100X, Bar 100 μm; *C*, H.E. 40X, Bar 100 μm) and **(D)** replacement with connective tissue (arrow) (Trichrome Mallory 40X, Bar 100 μm). **(E)** Regenerating myofibres were present within or near areas of degeneration, histologically characterized by the presence of rows of nuclei of internal myoblasts with bulky nuclei and prominent nucleoli (arrow) (P.A.S. 40X, Bar 100 μm).

Histochemistry and immunohistochemistry evaluations were performed on slow-twitch *Soleus* (SOL) (*n*. muscles = 2) and fast-twitch *Gastrocnemius* (GA) (*n*. muscles = 2), *Tibialis* (TA) (*n*. muscles = 2) and *Extensor digitorum brevis* (EDL) (*n*. muscles = 2) skeletal muscles. Hematoxylin-Eosin (H.E.), Mallory trichrome and P.A.S. stains showed marked fiber atrophy in all muscles, with a cross-sectional area (CSA) being markedly reduced (by 21.6%) in slow-twitch SOL muscle (Figures 6A,B,G). The diameter of succinodehydrogenase-stained fibers with high mitochondrial SDH activity, in SOL sections from Kir6.1^wt/VM^ mice, was also ∼20% less than in similar sections from WT mice. Lesions, histologically apparent as regressive-necrotic with regeneration zones, were present in SOL sections from Kir6.1^wt/VM^ mice. Advanced degeneration and regeneration, and replacement with connective tissues were observed in some groups of myofibrils using Mallory trichrome, but were P.A.S. negative (Figures 6C,D; Figures 7A–E). Myofibrils with angular atrophy and pyknotic nuclei were found (Figure 6D).

Atrophic phenomena were particularly evident in type I fibers of Kir6.1^wt/VM^ mice, but less in type II fibers, as observed with high myosin-ATPase activity at acidic pH and high mitochondrial SDH activity evidenced in muscle cryo-sections of GA muscle (Figures 6E,F). CSA was only reduced by 13.7% in fast-twitch GA muscle (Figure 6G); TA and EDL muscles were also less affected than SOL muscle.

**FIGURE 8:**
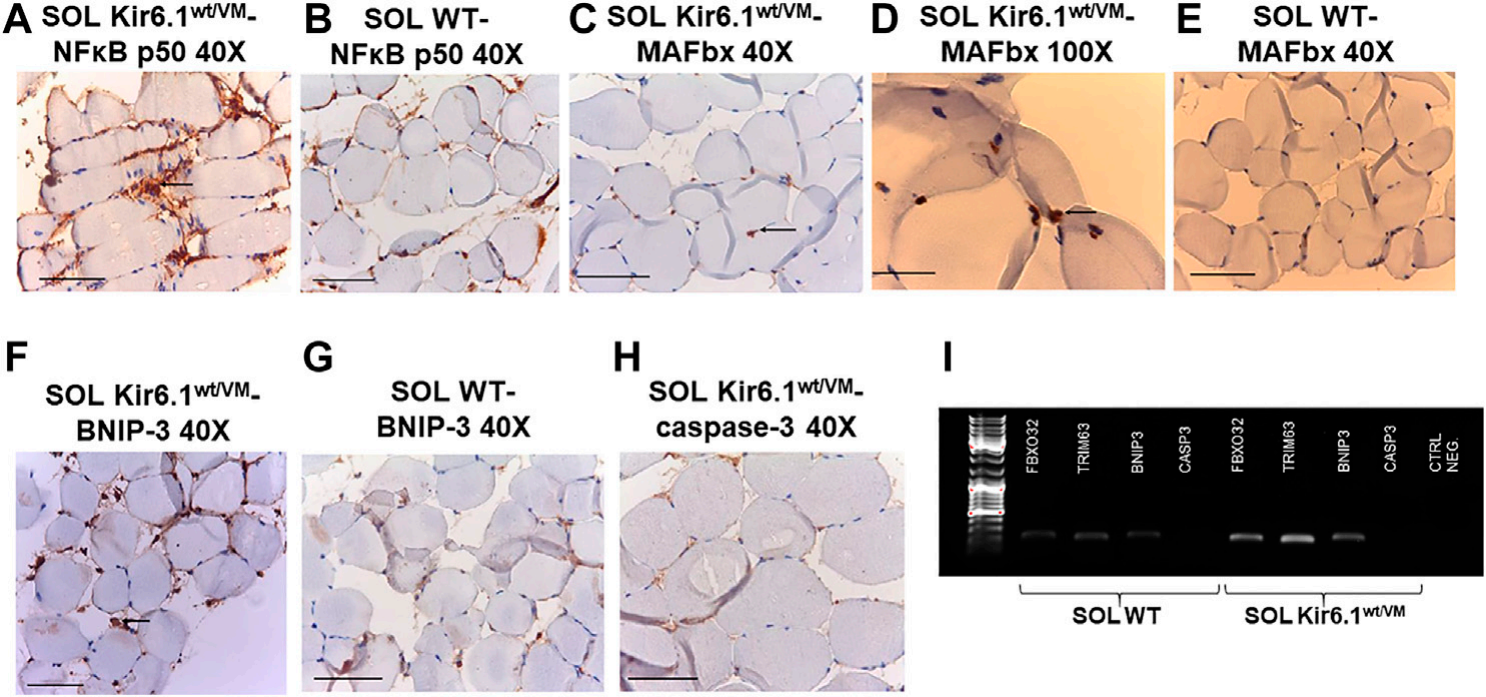
Immunohistochemistry analysis of slow-twitch SOL muscle from WT and Kir6.1^wt/VM^ mice. **(A)** Immunostaining (IMS) for NFκB p50 (E-10) on endomysia in SOL section from Kir6.1^wt/VM^ (arrow) (40X, Bar 100 μm), **(B)** negative reaction in WT mice (40X, Bar 100 μm). **(C,D)** Nuclear IMS for MAFbx (F-9) in the myofibrils of the Kir6.1^wt/VM^ mice (*C*, 40X, Bar 100 μm; *D*, 100X, Bar 100 μm); **(E)** negative IMS in the myofibrils of the WT mice (*E*, 40X, 100 μm). **(F)** IMS for BNIP-3 (ANa40) on endomysia in SOL section from Kir6.1^wt/VM^ (40X, Bar 100 μm); **(G)** negative IMS in the WT mice section (40X, Bar 100 μm). **(H)** Negative IMS for caspase-3 (E-8) in the Kir6.1^wt/VM^ mice section (40X, Bar 100 μm). **(I)** Representative sample gel of PCR analysis: in SOL muscle, *FBX O 32*, *TRIM63* and *BNIP3*, genes encoding atrogin-1, MuRF1 and BNIP3, are markedly up-regulated in Kir6.1^wt/VM^ SOL muscle. *Casp3* is not detected in SOL from both phenotypes.

Finally, immunohistochemistry was performed on sections of SOL, the most affected muscle in histological analysis, on silanized slides using anti-mouse primary antibodies for NFκB p50 (E-10), MAFbx (F-9), caspase-3 (E-8) and BNIP-3 (ANa40), which are respectively involved in inflammation, atrophy, apoptosis and autophagy processes in skeletal muscle. SOL sections of Kir6.1^wt/VM^ mice stained markedly positive for NFκB p50 and BNIP-3 (Table 3), with immunoreaction of inflammatory cells of the endomysium, compared to much lower positive staining in WT sections. Marked nuclear immunostaining (IMS) for MAFbx (F-9) was detected in Kir6.1^wt/VM^ but not in Table 3 WT sections (Figures 8A–H). Caspase-3 (E-8) was not detected in Kir6.1^wt/VM^ or WT sections (). PCR analysis revealed that *FBX O 32, TRIM63* and *BNIP3* (encoding atrogin-1, MuRF1, and BNIP3, respectively) are markedly up-regulated in Kir6.1^wt/VM^ SOL muscle; no expression of *Casp3* gene was detected in WT or Kir6.1^wt/VM^ muscle (Figure 8I).

**TABLE 3:** Immunohistochemistry on silanized slides using anti-mouse primary antibodies for NFκB p50 (E-10), MAFbx (F-9), caspase-3 (E-8) and BNIP-3 (ANa40) in soleus muscle sections from Kir6.1wt/VM and WT mice. Values are expressed as the percentage of immunostained cells. *Data significantly different with respect to the WT data (Student t test, *p* < 0.05).

Muscle types	NFκB p50		BNIP-3		MAFbx		Caspase 3	
	WT	Kir6.1^wt/VM^	WT	Kir6.1^wt/VM^	WT	Kir6.1^wt/VM^	WT	Kir6.1^wt/VM^
SOLEUS	16.6 ± 7%	*94.5 ± 10%	10.7 ± 6%	* 70.3 ± 11%	9.2 ± 5%	*40.1 ± 9%	7 ± 2%	n.s. 5 ± 1%

**FIGURE 9:**
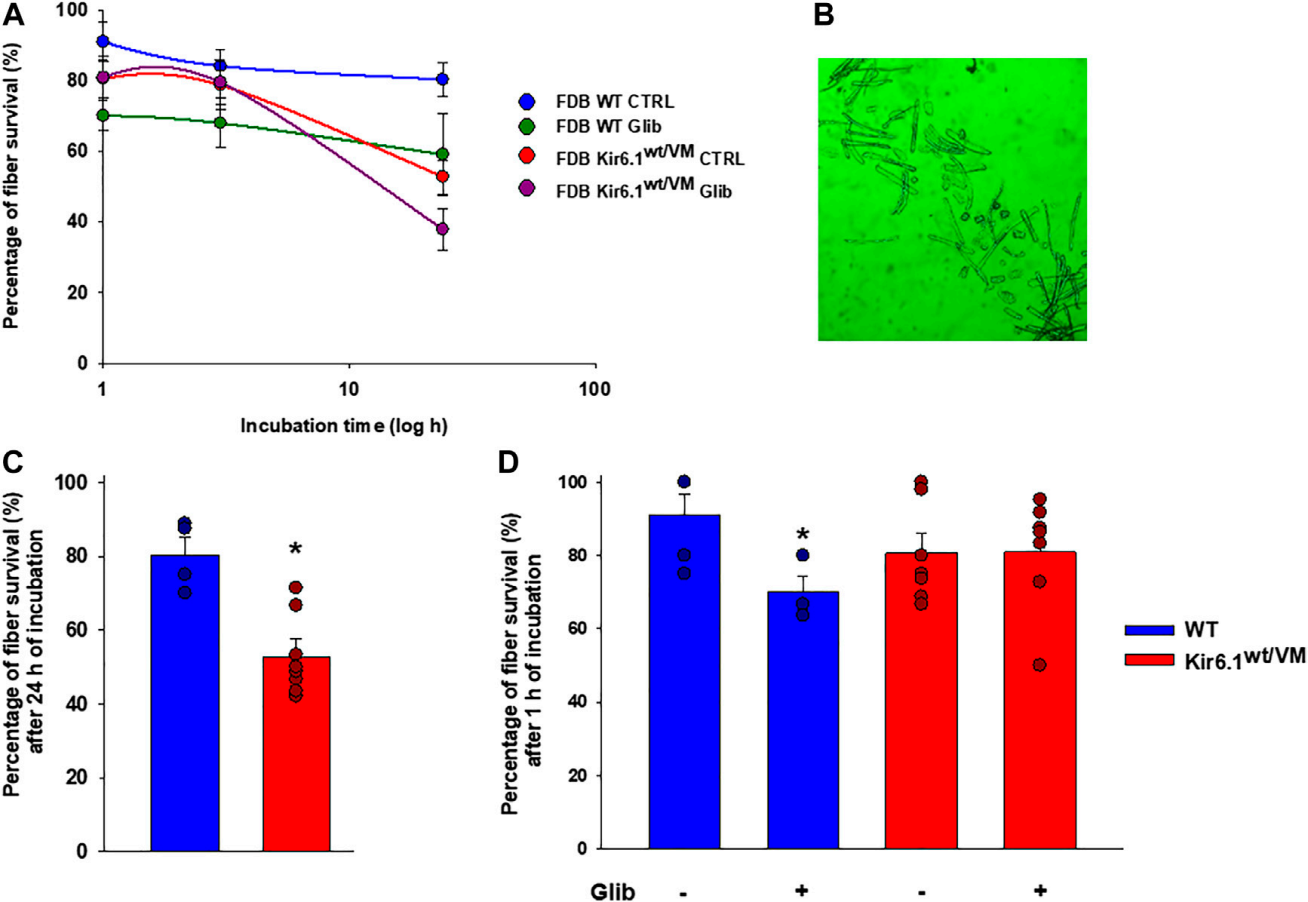
Immunohistochemistry analysis of slow-twitch SOL muscle from WT and Kir6.1^wt/VM^ mice. (A) Immunostaining (IMS) for NFκB p50 (E-10) on endomysia in SOL section from Kir6.1^wt/VM^ (arrow) (40X, Bar 100 μm), (B) negative reaction in WT mice (40X, Bar 100 μm). (C-D) Nuclear IMS for MAFbx (F-9) in the myofibrils of the Kir6.1^wt/VM^ mice (C, 40X, Bar 100 μm; D, 100X, Bar 100 μm); (E) negative IMS in the myofibrils of the WT mice (E, 40X, 100 μm). (F) IMS for BNIP-3 (ANa40) on endomysia in SOL section from Kir6.1^wt/VM^ (40X, Bar 100 μm); (G) negative IMS in the WT mice section (40X, Bar 100 μm). (H) Negative IMS for caspase-3 (E-8) in the Kir6.1^wt/VM^ mice section (40X, Bar 100 μm). (I) Representative sample gel of PCR analysis: in SOL muscle, *FBXO32, TRIM63* and *BNIP3*, genes encoding atrogin-1, MuRF1 and BNIP3, are markedly up-regulated in Kir6.1^wt/VM^ SOL muscle. *Casp3* is not detected in SOL from both phenotypes.

Besides, following isolation, *ex-vivo* survival over 24 h was worse for Kir6.1^wt/VM^ FDB fibers than for WT FDB fibers (Figures 9A,B). Although long-term incubation for 24 h in the presence of 10^−7^ M Glib significantly reduced fiber viability in both phenotypes (Figure 9C), Glib only caused a marked short-term loss of viability of WT fibers; consistently with the lack of inhibitory effect of Glib on Kir6.1^wt/VM^ mutant channels, no effect of Glib was observed on Kir6.1^wt/VM^ fiber survival (Figure 9D).

## Discussion

### KATP Channels and Skeletal Muscle

KATP channels are present in all muscle types and are abundant in skeletal muscle-sarcolemma (Tricario and Conte Camerino, 1994). Although gene knockout studies have implicated Kir6.2 and SUR2A as primary constituent subunits in most muscles (Spruce et al., 1985; Tricarico et al., 2006; Tricarico et al., 2016a), expression of all four KATP channel subunits has been demonstrated at the transcript level; and prior electrophysiological and pharmacological analyses provide evidence for expression of channels with single-channel conductance and drug sensitivities consistent with Kir6.1 and SUR1 expression contributing to channels with variable properties in different muscle types to overcame different muscle function. A straightforward hypothesis is that muscle KATP channels should open as the [ATP]/[ADP] falls during repetitive contractions, leading to hyperpolarization and action potential failure, thereby limiting contractile force in different skeletal muscle types. This would suggest that removing KATP channels would then enhance or prolong contractility. Kir6.2 knockout mice revealed rapidly fatiguing, weaker muscles (Cifelli et al., 2007), indicating that fatigue, and ultimately necrosis (Thabet et al., 2005), resulting from [Ca]-overload and energy depletion, as a consequence of the failure of KATP to limit excitability (Scott et al., 2016), is the dominant outcome.

Loss-of-function mutations in Kir6.2 and SUR1 are associated with congenital hyperinsulinism (Nestorowicz et al., 1996; Thomas et al., 1996; Dunne et al., 1997; Nestorowicz et al., 1997). Interestingly, there does not appear to be any obvious skeletal muscle defect, although Albaqumi et al. (2014) described a family with congenital hyperinsulinism and rhabdomyolysis associated with Kir6.2 mutations (Albaqumi et al., 2014), and Flanagan et al. (2017) recently reported a patient with persistent hyperinsulinaemic hypoglycaemia and severe hypotonia resulting from an activating mutation in a calcium channel subunit, which etiologically may have the same basis (Flanagan et al., 2017). Recently, we described a novel ABCC9-related Intellectual disability Myopathy Syndrome (AIMS) resulting from loss-of-function mutations in *ABCC9* (SUR2) (Smeland et al., 2019), in which patients also exhibit muscular pain and fatigue, and evidence of muscle fiber damage.

Conversely, increased KATP channel activity would be predicted to limit action potential generation, resulting in ‘electrical fatigue’ before energetic fatigue. Gain-of-function mutations in Kir6.2 and SUR1 are associated with developmental delay, epilepsy, and neonatal diabetes (DEND) syndrome, which is accompanied by muscle flaccidity and motor impairment. Transgenic mice with tissue-specific expression of Kir6.2 GOF in skeletal muscle have not revealed any obvious muscle phenotype (Clark et al., 2010), but there are no knock-in models of DEND with the targeting of the endogenous locus. Gain-of-function mutations in the other canonical KATP channel gene pair, Kir6.1, and SUR2, are associated with Cantù Syndrome (CS), with a distinct, unique, set of features. Among them, CS patients tend to have a muscular appearance, accompanied by hyperextensible joints and self-reported fatigue-ability.

In the present work, we studied the effects of the Kir6.1[V65M] mutation in slow-twitch and fast-twitch muscles of a new murine model of CS, in which patient-specific disease mutation is introduced to the identical locus in the mouse genome (Huang et al., 2018). *In vivo* experiments showed that Kir6.1^wt/VM^ mice generate significantly lower forelimb forces than WT mice. This is consistent with prior studies showing that abnormal activation of skeletal muscle KATP channels is associated with early fatigue during tetanus and that *in vivo* treatment of mice with KATP openers causes fatigue (Cifelli et al., 2008; Tricarico et al., 2016b), and also it resembles the fatigability and exercises intolerance observed in CS patients. Despite this weakness, isolated Kir6.1^wt/VM^ muscles tended to be heavier and, also, ultrasonography has revealed a significant increase of echodensity in Kir6.1^wt/VM^ hind limb muscles, associated with a tendency toward increased hind limb volume and vascularization. Additionally, histological analysis has revealed marked myofiber atrophy (see below). Taken together, these data suggest the presence of damage in skeletal muscle integrity along with fibrous tissue deposition (Pillen et al., 2009), explaining the increase in echodensity, the decrease of muscle strength, and the pseudo-hypertrophy.

### The Cellular and Pharmacological Consequence in Cantù syndrome Muscle

We, therefore, found an enhancement of the KATP current density in Kir6.1^wt/VM^ FDB fibers concerning WT and no significant enhancement of this parameter in SOL fibers of Kir6.1^wt/VM^. Patch clamp data allow statement that the Glib data indicates that mutant channels are present in the muscle.

The relative expression of different KATP channel subunits varies between muscles, and may be altered in different disease states (Tricarico et al., 2006; Tricarico et al., 2008b), though as yet unknown mechanisms. We do not have evidence of an increase of the Kir6.1 or Kir6.2 gene expression in the transgenic mice muscles, but our PCR analysis revealed expression of Kir6.1 subunit in WT SOL and FDB muscle but, unexpectedly, lower levels of Kir6.1 in Kir6.1^wt/VM^ FDB than in WT, despite higher KATP currents in the former. Reduction in mRNA expression, and of protein expression, has been observed for mutations linked to Kir6.2 GOF in neonatal diabetes (Lin et al., 2006; Lin et al., 2013), and we speculate that down-regulation of the over-activated Kir6.1 subunit in fast-twitch FDB muscle fibers could be a protective mechanism which preserves the function and the morphology of these tissues, with reduction of current density acting as a compensatory effect for the nucleotide insensitivity caused by mutation. Interestingly, there was no down-regulation of Kir6.1 transcript in Kir6.1^wt/VM^ SOL muscle, which additionally manifested worse atrophy, the low survival rate of the fibers, and lack of response to glibenclamide.

We however failed to show a significant rightward shift of MgATP-sensitivity in FDB and SOL patches. Based on experiments with recombinant channels, the loss of nucleotide inhibitory sensitivity is expected to be relatively subtle (Cooper et al., 2017) and can be masked in our experiment in native tissues due to the high expression levels of Kir6.2 and SUR2 subunits. The modest reduction of Glib-sensitivity observed in both Kir6.1^wt/VM^ FDB and SOL patches, as is reported for vascular smooth muscle cells in these animals (Huang et al., 2018), supports the presence of the Kir6.1 subunit in the muscle. It has been reported that the reduction in ATP sensitivity of Kir6.2/Kir6.1^(V65M)^ channels is ∼5 fold but the reduction of Glib sensitivity is ∼1000 fold heterologous expression system (Cooper et al., 2017). The 10 fold change in Glib sensitivity in muscle suggests is significantly less than that shown in heterologous expression. This also suggests that the reduction in ATP sensitivity of the KATP channel of the skeletal muscle is also drastically less than that the 5 fold change shown in heterologous expression (Cooper et al., 2017), and questions the pathological significance of its presence in the skeletal muscle. Additional experiments at single-channel levels are needed to clarify these issues.

The functional presence of a KATP channel current component uncoupled to the nucleotide metabolism as is expected for the Kir6.1^wt/VM^ mice may have deleterious effects specifically in the slow-twitch oxidative SOL fibers rather than in FDB or other fast-twitch muscles.

In WT FDB fibers, Glib induced cytotoxic effects within the first few hours of incubation but was without effect in Kir6.1wt/VM cells, consistent with loss of sensitivity to the drug. After long-term (24 h) incubation Glib had similar toxicity in both genotypes. Previous studies have demonstrated that *ex vivo* long-term exposure to KATP channel inhibitors is coupled to apoptosis and atrophic signaling in muscle fibers (Tricarico et al., 2010; Mele et al., 2014a; Mele et al., 2014b; Cetrone et al., 2014), although this may be due to non-KATP channel-dependent actions of the drug (de Sant’Anna et al., 2015; Subramaniyam et al., 2018). We have recently shown that Glib treatment can effectively reverse many of the cardiovascular complications of CS (Ma et al., 2019; McClenaghan et al., 2020). However, reduced Glib sensitivity in recombinant Kir6.1[V65M] channels, and the markedly reduced sensitivity that we show here, raises the potential that Glib treatment may be ineffective for treating muscle impairments in certain patients and that other therapeutic approaches may be needed.

Atrophy was more marked in Type I fibers than Type II, and there was a markedly lower survival rate in Kir6.1^wt/VM^ SOL vs. FDB fibers. This raises the possibility that SOL muscle, and slow-twitch fibers more generally, maybe more severely affected in CS than fast-twitch fibers, such as FDB. Markers of atrophy and autophagy, including *Atrogin-1, MuRF1,* and *BNIP3* genes were all upregulated in Kir6.1wt/VM SOL, and expression was not detected in WT muscles (Foletta et al., 2011). Relative to WT, muscle diameter was more markedly reduced in Kir6.1^wt/VM^ SOL than fast-twitch GA muscle, further suggesting a major pathological involvement of slow-twitch fibers, a possibility that should be examined in future studies of CS patients.

An additional hypothesis can be drawn explaining at least in part the observed changes of the muscle function in the Kir6.1^wt/VM^ mice. We have shown that changes in heart size/function in the CS mouse model are secondary to changes in the KATP channel function in Vascular Smooth Muscle and these changes are reversed by glibenclamide (McClenaghan et al., 2020). The recorded changes in muscle function may be therefore secondary to skeletal muscle remodeling rather than an altered function of the KATP channel in the SOL muscle as observed in the cardiovascular apparatus in these mice. It should be of note, however, that we failed to evidence any significant change of % of vascularization of the hind limb muscles in the Kir6.1^wt/VM^ mice vs WT mice as evaluated by ultrasonography thereby not supporting this hypothesis. Inflammatory cells were also observed in CS muscle. The evaluation of the relative contribution of the direct and indirect effects of the Kir6.1^wt/VM^ mutation in skeletal muscle requires further experiments.

This is the first report showing a direct genotype-phenotype correlation in CS skeletal muscle, revealing how Kir6.1^wt/VM^ mutation in the KATP channel Kir6.1 subunit is associated with a reduction of limb strength, skeletal muscle atrophy, autophagy, and connective tissue replacement of myofibers in this animal model of CS. It should of note that several factors can mediate the muscle-specific degeneration that we found in Kir6.1^wt/VM^ SOL muscle. For instance, lactate accumulation into the muscle following the overactive KATP channels may be involved in atrophy and degeneration in slow-twitch muscle.

In conclusion, these data suggest that Kir6.1^wt/VM^ mutation affects directly and/or indirectly skeletal muscle through vascular dysfunction and this could be a significant issue, particularly for slow-twitch muscle, in CS patients suffering from this mutation and warrants future investigation.

## Data Availability Statement

The raw data supporting the conclusions of this article will be made available by the authors, without undue reservation, to any qualified researcher.

## Ethics Statement

The animal study was reviewed and approved by Animal care and all experimental protocols are in accordance with the European Directive 2010/63/EU on Animal Protection Used for Scientific Experiments, and the Washington University School of Medicine Institutional Animal Care and use Committee, and were approved by the Italian Ministry of Health and by the Committee of the University of Bari O.P.B.A (Organization for Animal Health) (prot. 8515-X/10, 30-01-2019).

## Author Contributions

CM and TH was responsible for animal husbandry, DT and CN wrote the manuscript, RS contributes significantly to data presentation and analysis. GC and RS performed PR experiments. RS and FM performed patch-clamp and *in vitro* experiments on native fibers, and organs and tissues isolation. AM was responsible for ultrasonography of hind-limb muscle evaluation. NZ was responsible for mice health. NZ and GR performed histochemistry and immunohistochemistry experiments. FZ was responsible for immunohistochemistry data. All authors critically reviewed the manuscript.

## Funding

This work was supported by NIH R35 HL140024 (to CN). CM was supported by American Heart Association Postdoctoral Fellowship 19POST34380407. This research was also supported by M.I.U.R. PhD program to RS (tutor DT) and in part funded by Regione Puglia (Italy) project “Cluster in Bioimaging” code QZYCUM0, through FSC 2017-2013/Programma regionale a sostegno della specializzazione intelligente e della sostenibilita ambientale. Intervento “Cluster Tecnologici Regionali (DT).

## Conflict of Interest

The authors declare that the research was conducted in the absence of any commercial or financial relationships that could be construed as a potential conflict of interest.
